# 肺癌演进与癌相关成纤维细胞代谢转变的关系及进展

**DOI:** 10.3779/j.issn.1009-3419.2014.09.07

**Published:** 2014-09-20

**Authors:** 恒 杜, 国卫 车

**Affiliations:** 610041 成都，四川大学华西医院胸外科 Department of Toracic Surgery, West China Hospital, Sichuan University, Chengdu 610041, China

**Keywords:** 癌相关成纤维细胞, 代谢转变, 肺肿瘤, Cancer-associated fbroblasts, Metabolic alteration, Lung neoplasms

## Abstract

肺癌的演进与“肿瘤微环境”变化密切相关，癌相关成纤维细胞（cancer-associated fibroblasts, CAFs）是被癌细胞“驯化”的成纤维细胞，是肿瘤微环境的重要成员之一。CAFs还具有促进肿瘤细胞生长、侵袭和转移的特性。研究表明CAFs的物质能量代谢方式与正常的成纤维细胞有明显不同。CAFs以糖酵解生成乳酸的方式进行代谢并将乳酸供给癌细胞，即CAFs表现为“反瓦伯格效应（reverse Warburg effect）”的代谢形式以适应和促进肿瘤细胞的演进。本文针对CAFs代谢转变与肺癌演进的关系，从以下五个方面进行综述：①CAFs的特性及其代谢特点；② CAFs代谢的研究现状；③CAFs代谢方式转变可能的分子机制；④CAFs代谢方式转变与肺癌演进的关系；⑤CAFs代谢方式的转变与肺癌预后和治疗的关系。

## Abstract (en)

Tumor microenvironment is closely related to tumor progression. Cancer-associated fbroblasts (CAFs) are specialized fbroblasts domesticated by cancer cells surrounding them. CAFs are one of the most important components in cancer microenvironment. CAFs extensively involved in tumor genesis, tumor invasion and metastasis. Compared to NFs, CAFs also have a different metabolism type. CAFs use glycolysis to produce lactate for cancer cells. Tat is, CAFs turn to a metabolism type named "reverse Warburg effect" to promote tumor progression. In the present study, we summarized that: (1) Characteristics and metabolic type of CAFs; (2) The research status of the metabolism of CAFs; (3) The potential mechanism of CAFs metabolism; (4) The relation between metabolic alteration in CAFs and lung cancer progression; (5) Relationship between CAFs metabolism and lung cancer prognosis and therapy.

肿瘤的生长与肿瘤细胞所处的内外环境有着密切关系，其中肿瘤微环境发挥了重要作用。肿瘤微环境指肿瘤细胞周围的免疫细胞、间质细胞及其分泌的细胞因子等与肿瘤细胞相互作用而构成的局部内环境。肿瘤微环境中的主要细胞成分包括癌相关成纤维细胞(cancerassociated fibroblasts, CAFs)、癌相关巨噬细胞、肿瘤微血管及淋巴管、炎症细胞及免疫细胞等。CAFs作为肿瘤微环境中的主要部分，不仅通过分泌多种细胞因子与癌细胞之间存在产生广泛的交互对话(cross-talk)，而且在促进肿瘤细胞增殖、移动和远处转移，维持肿瘤微环境慢性炎症状态易化癌细胞转移，肿瘤微血管形成等方面也扮演十分重要的角色^[1-3]^。肿瘤细胞的快速增殖与生长是以大量的物质和能量代谢为基础，癌细胞及其"微环境"的物质供应和能量代谢必然以不同于正常细胞的代谢方式来进行，以此适应癌细胞不受调控的快速生长及侵袭转移^[4, 5]^。经典的瓦伯格效应(Warburg effect)认为癌细胞即使在氧供充足的情况下仍以糖酵解生成乳酸的方式进行物质能量代谢^[6]^。近来也有研究^[7-10]^发现CAFs具有独特的代谢方式，即CAFs通过糖酵解方式将摄取的葡萄糖转化为乳酸，供给其周围的癌细胞，而癌细胞则直接用乳酸进行三羧酸循环(tricarboxylic acid cycle, TCA)。由癌细胞诱导、氧化应激造成的CAFs "线粒体自噬" (mitophagy)是CAFs代谢方式改变的基础。癌细胞与微环境中CAFs物质与能量代谢方式转换的相互适应，使癌细胞的营养物质及能量来源主要直接依赖于CAFs而不依赖于肿瘤微血管。癌细胞能量来源的变化可能是目前针对"种子"(肿瘤细胞)治疗效果差(如化疗药物耐药)及抗血管药物(血管内皮生长因子抑制剂，如贝伐珠单抗)短期有效而长期耐药的主要原因^[11, 12]^。研究CAFs代谢方式转换的分子机制，有助于我们更好地了解肿瘤的生长环境，并有望成为肿瘤治疗的新策略。

## 1 CAFs的特性及代谢特点

CAFs是癌组织基质中呈激活状态的成纤维细胞^[13, 14]^。相比于正常的成纤维细胞(normal fibroblasts, NFs)，CAFs具有如下几个方面的不同：①CAFs细胞的形态呈大小不均匀的纺锤形，排列无方向性，胞质内含肌丝，而NFs一般为大小一致，排列整齐的星形，其胞质内不含肌丝但有丰富的高尔基体；②CAFs的分子标志表现为高表达α-平滑肌动蛋白(α-smooth muscle actin, α-SMA)、成纤维细胞活化蛋白(fibroblast activation protein, FAP)、波形蛋白(Vimentin)，而上皮性钙粘附因子(E-cadherin)、细胞角蛋白-19(cytokeratin-19, CK-19)、CD31等上皮性标志低表达甚至不表达^[1, 13, 15]^；③CAFs能通过不同的途径促进癌细胞的增殖与转移。与NFs抑制癌细胞的作用相反，CAFs则能通过Ark和Erk途径、或分泌IL-6、IL-8等炎性因子促非小细胞肺癌(nonsmall cell lung cancer, NSCLC)增殖、侵袭和转移^[16, 17]^。除了IL-6、IL-8，CAFs还与多种炎症因子相关^[18]^，CAFs介导的肿瘤微环境慢性炎症状态能易化癌细胞的远处转移^[19]^。除了上述细胞因子途径，CAFs还可通过SDF-1/CXCL -12、IL -6、及P16INK4A等不同途径促进癌微环境中血管形成而间接发挥促进癌细胞增殖和转移的作用^[3, 20, 21]^。此外，有研究^[1]^发现单独注射CAFs于免疫缺陷的小鼠不会形成肿瘤，这表明CAFs自身并无成瘤能力。另一些研究^[21-23]^表明，肺腺癌对靶向治疗药物(目前主要是吉非替尼)产生耐药性被认为可能与上皮-间质转换(epithelial- mesenchymal transition, EMT)及通过该过程形成的CAFs有关。

较之正常的成纤维细胞，CAFs的代谢方式也具有特殊性。有研究^[8, 24]^认为CAFs与癌细胞的代谢偶联方式更符合于"反瓦伯格效应"，具体表现为：在周围癌细胞的氧化应激的作用下，CAFs的线粒体破坏并被溶酶体清除，迫使CAFs使用糖酵解的方式进行代谢，产生的乳酸盐被癌细胞膜高表达的MCT1(一种将乳酸转入细胞内的膜蛋白)摄取，摄入的乳酸盐进入癌细胞线粒体内参与TCA，为癌细胞的生长提供能量^[8]^。其中CAFs产生的乳酸盐还能刺激癌细胞的线粒体发生和氧化磷酸化反应。因此，氧化应激导致的CAFs线粒体破坏(线粒体自噬)是其代谢方式改变的原因^[25]^，免疫组化染色的结果^[11]^也证实癌组织基质内Bnip3L、ATG16L、Cathepsink A和D等线粒体损伤标志物呈现强阳性表达。

CAFs的代谢方式的改变还具有"场效应"(field effect)的特点，即CAFs能将其自身代谢方式的改变传播给周围正常的成纤维细胞。有关"场效应"的机制目前还不清楚，可能是通过癌组织中广泛存在的癌相关炎症反应的微环境产生上述作用^[9]^。

## 2 CAFs代谢的研究现状

自20世纪30年代Warburg提出癌细胞代谢的"瓦伯格效应"以来，大量研究主要集中在针对癌细胞代谢方式上。在PubMed中以"Warburg effect [Title]"检索，最早的文献为1969年。以"(cancer cells [Title]) AND metabolism [Title]"为检索式，截至2014年3月31日，共命中272篇文献。而以"(cancer associated fibroblasts[Title]) AND metabolism[Title]"检索，仅命中3篇文献。为避免检索式设计造成的文献检索遗漏，对上述3篇文献所列出的参考文献进行复习，并根据相关参考文献的作者信息等进行手工检索，共得到与CAFs代谢相关的文献36篇，最早的文献发表于2006年。说明CAFs的代谢特点近几年才逐渐被认识到。针对CAFs代谢机制的研究则主要集中于乳腺癌，而其他肿瘤(如肺癌、肝癌、结直肠癌等)则研究相对较少。目前研究的共识可概括为"反瓦伯格效应"即癌细胞和周围CAFs存在的共同演化(co-evolving)和代谢偶联(metabolic coupling)现象。

## 3 CAFs代谢方式转变可能的分子机制

### 3.1 转化生长因子β(transforming growth factor β, TGF-β)信号途径

TGF-β广泛参与到炎症、肺纤维化、肺癌等多种疾病当中并对疾病的发生发展起到十分重要的作用^[26, 27]^。研究发现TGF-β在癌的发生演进中扮演"双刃剑"的角色，即在肿瘤起始时抑制肿瘤细胞的生成，随后则促进肿瘤的侵袭和转移。Zavadil等^[28]^研究表明，TGF-β通过促进EMT过程，进而促进癌细胞的生长和转移。此外，有研究^[11, 29]^发现：配体依赖的(ligand dependent)或细胞分泌的TGF-β能改变CAFs的代谢方式，上调CAFs中低氧诱导因子-1α(hypoxia-inducible factor-1α, HIF-1α)的表达。HIF- 1α ^[30]^是一种转录因子，能介导细胞对低氧和氧化应激的应答。由于CAFs中氧化应激增强、线粒体功能受损(表现为OXPHOS表达上调)，线粒体自噬增强(表现为Bnip3L表达增强)、糖酵解生成增加产生大量乳酸盐，同时还由于TGF-β诱导上调的CAFs膜蛋白MCT4，从而使胞内的乳酸盐排出增多，为癌细胞的生长提供"燃料"。有研究表明TGF-β的作用还具有细胞特异性即来源于CAFs自分泌或癌细胞旁分泌的TGF-β必须作用于CAFs细胞才能发挥作用，单独激活癌细胞膜上的TGF-β受体对癌细胞的生长不产生影响。

### 3.2 小窝蛋白(Caveolin-1, Cav-1)信号途径

Cav-1一直被认为具有抑制癌症的作用^[31]^，其低表达是乳腺癌预后差的独立危险因素，与肿瘤的早期复发、淋巴结转移以及他莫昔芬耐药等均有关。多项研究^[7, 32]^均表明Cav-1的低表达与CAFs的代谢方式的改变具有密切的关系。引起Cav-1下调的因素有很多，*H-Ras*突变、*p53*突变、cMyc高表达均可导致其表达下调。而CAFs中的Cav-1下调的根本原因是氧化应激。

癌细胞介导周围的CAFs产生氧化应激，激活后者的HIF-1α途径和核转录因子(nuclear factor κB, NF-κB)途径，通过溶酶体自噬作用使得Cav-1下调。使用小干扰RNA(small interfering RNA, siRNA)干扰Cav-1的表达发现Cav-1的急性下调导致CAFs中的线粒体损伤和线粒体自噬作用增强，结果是CAFs糖酵解增加，产生乳酸盐增加，从而支持癌细胞的无限生长。Martinez-Outschoorn将癌细胞和CAFs共培养后，发现两者的过氧化物还原酶-1 (peroxiredoxin-1)的活性均增加，这可能是两者在氧化应激环境下的自身保护机制之一。然而活性氧(reactive oxygen species, ROS)的明显增多使得CAFs中DNA双链损伤(免疫组化表现为γ-H2AX染色强阳性)，但在CAFs中并未表现出明显的凋亡迹象。因此推测两者除了peroxiredoxin-1外，还有其他的抗凋亡的机制。此外有研究^[33]^显示通过激活癌细胞中的HIF-1α途径，也可以增强癌细胞的自噬作用，使癌细胞的生长受到明显抑制。

## 4 CAFs代谢方式转变与肺癌演进的关系

CAFs代谢方式的改变在肿瘤细胞的生长、移动、侵袭和转移过程中发挥十分重要的作用。肺癌细胞产生的氧化应激通过上述途径造成周围的CAFs发生自噬和线粒体自噬(autophagy/mitophagy)^[9]^。CAFs的自噬产生了氨基酸、脂肪酸核苷酸等产物，这些产物均可以提供给周围的癌细胞使用。其次，由CAFs的线粒体自噬后的糖酵解反应所产生的大量乳酸源源不断地输送给癌细胞，相较于使用葡萄糖，肺癌细胞直接利用呼吸链中的半成品——乳酸进行TCA从而更快捷地获取养料和能量^[34, 35]^。乳酸还能刺激癌细胞中的线粒体活性，使之数量增加、诱导产生氧化应激的能力增强，CAFs接受氧化应激后又重复自噬，由此产生恶性循环^[36]^。CAFs代谢方式的改变除了为癌细胞的生长提供能量直接促进癌细胞生长外，还能抑制癌细胞的凋亡。CAFs能通过促进周围癌细胞中p53介导的糖酵解和凋亡控制因子(Tp53-induced glycolysis and apoptosis regulator, TIGAR)高表达，而使癌细胞免除自身受到氧化应激损伤，同时也使其逃脱凋亡^[37, 38]^。CAFs还介导癌细胞中的基因不稳定性，通过"bystander"效应增加癌细胞的攻击性^[7]^。肺癌中以糖酵解为代谢方式的CAFs产生的乳酸和羟丁酸具有直接刺激癌细胞移动的能力，Bonuccelli等^[39]^的实验证明乳酸和羟丁酸能使癌细胞的移动速度增加2倍。

CAFs中Cav-1的下调导致肺癌基质的重塑，加之乳酸的堆积使得肺癌基质呈现弱酸性，在介导周围正常细胞凋亡的同时也使得基质水解，以有利于肺癌细胞的转移^[40]^。CAFs代谢改变相关的HIF-1α、NF-κB信号能促使尿激酶型纤溶酶原激活物(urokinase-type plasminogen activator, uPA)与相应受体结合，导致纤溶酶原激活为纤溶酶，继而激活基质金属蛋白酶(matrix metalloproteinase, MMP)，后者降解胞外基质蛋白，易化癌细胞的迁移^[41]^。研究^[42]^也显示了MMPs高表达与肺癌预后呈负相关。NF-κB是一个多效的信号通路，其在炎症中也发挥关键的作用。NF-κB能使得肿瘤微环境维持在慢性炎症状态，而微环境中的慢性炎症状态是促使癌细胞增殖、转移的另一重要原因^[14, 19]^。

## 5 CAFs代谢方式的转变与肺癌治疗和预后的关系

过去认为肺癌细胞的生长伴随着癌中血管的发生。然而抑制血管生成的化疗药物仅短期内有效且很容易引起肺癌复发和转移，CAFs的自噬现象则能够解释该现象^[43]^。其原因：一是癌细胞的能量来源于CAFs而不依赖新生血管；其次，血管内皮生长因子抑制剂由于抑制了肿瘤新生血管的生成，加重了肿瘤微环境的低氧状态^[44]^，更进一步刺激CAFs的氧化应激和糖酵解，从而产生更多的乳酸盐，促进癌细胞的生长和转移。

癌细胞"驯化"的CAFs中产生的一系列反应最终造成癌细胞和周围基质中的能量不平衡——"燃料"通过CAFs被源源不断地输送给癌细胞，基质的分解代谢成为癌细胞合成代谢的基石。上述过程中，肿瘤微环境中的"自噬"起到了十分重要的作用。如果将这种"自噬"放大至整个人体，则表现为癌相关的恶病质^[32, 45]^。癌组织通过增强机体的分解代谢进而为自己的生长和远处转移供能。肺癌患者晚期表现出的严重恶病质原因正基于此。因此，CAFs代谢方式改变造成的癌细胞扩散和恶病质给肺癌患者以双重致命打击。在肺癌预后的研究方面，发现CAFs的Cav-1下调预示着癌细胞的侵润和远处转移，与肺癌患者的预后成负相关^[46]^。

## 6 问题与展望

CAFs代谢方式改变的始动因素是氧化应激的产生和线粒体自噬。在实验研究过程中如果加入自噬抑制剂氯喹或者抗氧化剂，均可以使得CAFs中的Cav-1的表达升高，受损的线粒体功能恢复，CAFs的恶性表型被逆转^[11]^。另外，抗氧化剂对癌细胞的抑制作用也为肿瘤的药物治疗提供了新的策略。

目前关于CAFs代谢方式机制的研究主要集中于Cav-1通路，所用的模型也更多限于乳腺癌，针对肺癌的研究相对较少。现有文献的争议包括：①CAFs的特殊代谢方式是否涉及到遗传学的改变；②肿瘤微环境中的慢性炎症可能参与了CAFs恶性表型的传播，在炎症中是否也有相应的细胞代谢方式的改变；③人类的良性肿瘤相较于恶性肿瘤具有生长局限，无远处转移的特点，良性肿瘤中成纤维细胞是否仍维持正常细胞的代谢方式？④CAFs糖酵解所需的大量葡萄糖通过何种方式进入癌组织内从而被CAFs利用，是否也不依赖于肿瘤微血管。上述的问题有待于未来更多的研究来解答。
